# Chemical Composition of the “Galo de Barcelos” (Barcelos Rooster Raw Meat)

**DOI:** 10.3390/ani12121556

**Published:** 2022-06-16

**Authors:** Isabel Maria Afonso, Susana Casal, Júlio César Lopes, Jéssica Domingues, Ana Paula Vale, Márcio Meira, Maria Conceição Marinho, Pedro Santos Vaz, Nuno V. Brito

**Affiliations:** 1 CISAS—Center for Research and Development in Agrifood Systems and Sustainability, Polytechnic Institute of Viana do Castelo, 4990-706 Refóios do Lima, Portugal; iafonso@esa.ipvc.pt (I.M.A.); juliocesar@esa.ipvc.pt (J.C.L.); anapaula@esa.ipvc.pt (A.P.V.); 2 LEPABE—Laboratory for Process Engineering, Environment, Biotechnology and Energy, Faculty of Engineering, University of Porto, Rua Dr. Roberto Frias, 4200-465 Porto, Portugal; 3 LAQV@REQUIMTE/Faculty of Pharmacy, University of Porto, 4050-313 Porto, Portugal; sucasal@ff.up.pt; 4 Agrarian Higher School, Polytechnic Institute of Viana do Castelo, 4990-706 Refóios do Lima, Portugal; jessicadomingues@ipvc.pt (J.D.); marciomeira@ipvc.pt (M.M.); mariasusi@esa.ipvc.pt (M.C.M.); 5 Gastronomic Fraternity “O Galo de “Barcelos””, 4750-783 Barcelos, Portugal; psantosvaz@gmail.com; 6 TOXRUN—Toxicology Research Unit, University Institute of Health Sciences, CESPU, CRL, 4585-116 Gandra, Portugal

**Keywords:** “Barcelos” rooster, chemical, meat quality, controlled production, genotype

## Abstract

**Simple Summary:**

The assessment of traditional products is important for the sustainability of agricultural systems and the preservation of a unique gastronomic heritage. The present study aims to determine the chemical composition of “Galo de “Barcelos”” (“Barcelos” Rooster) raw meat, used in the preparation of the typical Portuguese dish “Roasted Rooster from “Barcelos””, in order to evaluate and protect this gastronomic and cultural tradition. The influence of the genotype on the final product was verified, concerning total protein, and fat contents, and a favorable ratio of n-6/n-3 fatty acids of the Sasso line was compared to the “Amarela” breed, contributing to the promotion of its gastronomic potential.

**Abstract:**

Ten roosters produced according to “Barcelos Confraria” rules and ten roosters of autochthonous “Amarela” breed, reared on a similar traditional production system, were analyzed, and the chemical profile of two of the most significant meat portions, breast and drumstick, was determined. The results demonstrated that the “Barcelos” rooster raw meat is rich in proteins (22.3%) and fat (4.31%), particularly in monounsaturated fatty acids (39.1%). Significant differences (*p* ≤ 0.01) were observed, with the breast having a higher protein content (25.1 vs. 19.7%) and less fat (1.9% vs. 6.7%), compared to the drumstick. The fatty acid profile revealed (SFA 30.0%, MUFA 39.1%, and PUFA 24.6%) a similar composition to the roosters reared in the traditional or organic production systems, such as the “Amarela” autochthonous rooster. The “Barcelos” rooster can be regarded as a highly nutritional meat, with an interesting chemical profile ensuring a high-quality traditional product to consumers.

## 1. Introduction

Meat is one of the most nutritious foods, and poultry meat is known for its nutritional quality as it contains a significant amount of high-quality proteins, is highly digestible, has a high content of vitamins and minerals, and has a low portion of saturated fat.

Chicken meat is highly recommended for all age groups, and its consumption has been privileged by consumers, causing a significant increase in its production [1,2]. In Portugal, in 2018, poultry meat consumption per capita (kg/inhabitant) was 42.8 kg/inhabitant/year, the second-most consumed meat, after pork (about 44.7 kg/inhabitant/year) [3].

A precise evaluation of meat physicochemical parameters is based on the determination of its nutritional composition. Meat characteristics, such as pH, moisture, fat, and fat profile or protein, are the most frequently determined parameters with traditional methodologies. However, studies [4] have been carried out to evaluate the efficiency of different non-destructive techniques based on images and/or spectra for the analysis of quality parameters of meat and meat products, as alternative and/or complementary to the traditional methods.

Meat quality is affected by different parameters, including intrinsic parameters such as age, sex, genetics, nutrition, and physiological state and extrinsic parameters such as the environmental and sanitary conditions, feeding techniques, management, transport, slaughter, and postmortem meat processing. Fat quality or nutrient quantities depend largely on the animal diet or its genetic potential, although the mode of production (organic and free range) influences some aspects of meat composition [5].

Consumers’ concerns about health and nutritional diet quality created a growing interest in poultry products from outdoor production systems [6,7]. The use of local breeds, closely related to the specific environments, contributes to the maintenance of biodiversity in a sustainable agriculture, especially in less favored areas. The rearing of these breeds under traditional methods plays an important social, economic, and cultural role in the aforementioned areas [8].

With the considerable growth in consumer concern about animal welfare in recent years, ‘organic’ animal breeding now presents an opportunity for more traditional production systems [9]. The market increasingly favors identity, genuineness, ethics, and sustainability, characteristics that are pillars of many traditional agrifood products and agricultural production methods.

The connection of the product to its region and the particularities of its manufacturing processes create new opportunities for farmers and local communities to associate the supply of services with production, which is the traditional products’ main value for their contribution to rural development [10].

Portugal has an extensive variety of traditional food products, strongly associated with the region and the cultural influence of its preparation, constituting a living, unique, and rich gastronomic heritage. Promoting local products and characterizing and guaranteeing authenticity are determining factors for their recognition and sustainability.

The “Roasted Rooster from Barcelos” is one of the most typical dishes of local gastronomy, with a strong connection to one of the most emblematic icons of the Portuguese cultural tradition. It is a gastronomic delicacy reminiscent of the Legend of the Rooster and brings to the table the ceremony, history, and symbolism of Santiago Compostela Way. It promotes a journey through the Jacobean tradition and the paths of knowledge and flavors of tradition [11]. The roasted rooster is the most important reference of the “Barcelos” cuisine and restaurants.

The “Confraria Gastronómica o Galo de Barcelos” (gastronomic fraternity) was created in 2016 with the purpose to value and promote the “Galo de Barcelos” (“Barcelos” Rooster) in its cultural and gastronomic aspects. The “Confraria” established a set of production rules, which included feeding, density, housing, and sanitary aspects [12]. To guarantee the traceability of the entire process, every animal was identified with a wing tag, as a serial number, enabling the entire production process to be controlled, from the chick delivery to the restaurant. The “Galo de Barcelos” specification’s production system implies the use of autochthonous breeds or slow-growth line roosters. The animals can only be fed with a specific diet formulated by the “Confraria”; this diet should include access to pasture, and have corn and vegetables, such as galega-cabbage (*Brassica oleracea*, var. *acephala*). These characteristics limit the potential growth of the animals but improve meat quality and organoleptic attributes.

The aim of this study is to provide the chemical characterization of the “Barcelos” rooster, identifying its particularities in relation to an autochthonous breed produced in a traditional system, in order to promote and qualify this important endogenous resource.

## 2. Materials and Methods

### 2.1. Sample Size

Twenty roosters were used; 10 of “Barcelos” (Sasso broiler genotype) and 10 of the autochthonous “Amarela” breed. “Barcelos” roosters originated from 6 exploitations, associated with the “Confraria”, in the region considered to be the breeding area (municipality of “Barcelos” and border villages). These farms are characterized by small effective and traditional production systems, in terms of housing and feed management. “Amarela” roosters originated from 6 exploitations; in the same geographic region and mode of production; with access to pasture; fed on corn and vegetables; and with an average slaughter age of 140 days and an average live weight of 3.5 kg. “Barcelos” roosters, identified with a wing tag, as a serial number, enabling the entire production process to be controlled, originated from slow-growth line roosters (Sasso liner). The animals comprising the sample were raised and kept by backyard producers under extensive conditions and were fed on a specific diet, according to “Confraria” rules, corn, and farm fodder, such as galega-cabbage (*Brassica oleracea*, var. *acephala*), which were complemented with surplus or by-products from human or animal feeding. The “Barcelos” roosters, at 140 days of age and 4.2 kg of bodyweight (BW), in accordance with “Confraria” production regulations, and the “Amarela” roosters at 3.5 kg average bodyweight, after fasting (cereal only, due to extensive production system) of 12 to 16 h, were slaughtered in a local plant, according to legislation rules [13]. The roosters were electrically stunned, killed by manual exsanguination, plucked, and weighed (weight of plucked and bled carcass (CW1)). After evisceration and carcass refrigeration (24 h at 4 °C), two other weight measurements were determined, corresponding to the eviscerated carcass weight with (CW2) or without head, neck, legs, and edible viscera (CW3), and, from breast and drumstick, the *pectoralis major* and *peroneous longus* muscles were excised for analysis. All procedures were conducted according to the guidelines of the organism responsible for the Animal Welfare of the Polytechnic Institute of Viana do Castelo—ORBEA-IPVC, in accordance with Decree-Law No. 113/2013 of 7 August.

### 2.2. Analytical Determinations

The chemical composition of the roosters’ breast and drumstick portions was determined. The meat samples were prepared in the laboratory, consisting of the removal of the skin and bones and then homogenization for 2 min (Grindomix GM200, Retsch, Haan, Germany). The parameters were determined in duplicate according to the AOAC [14].

The pH of the samples was measured using a digital portable pH-meter (FC2022/HALO^TM^, Hanna Instruments, Eibar, Spain) equipped with a penetration probe, according to the ISO 2917:1974 method [15]. Moisture, following the drying method up to a constant weight at 105 °C in a stove, was quantified according to the ISO recommended standard 1442:1997 [16]. Ash content was estimated via incineration (muffle B150, Nabertherm, Germany) of the samples, for 6 h and at a temperature of 550 °C ± 25 °C, according to ISO 936:1998 method [17].

Protein content was determined according to the Kjeldahl method (ISO 937:1978) [18], with the digestion (DK 20, Heating Digestor, Velp Scientifica), distillation (UDK 139, semiautomatic distillation V. Scientifica), and titration (Titroline 5000, SI Analytics) of samples, multiplying the amount of azote by a factor of 6.25. The sodium content was measured according to ISO 1841:1981 [19], converting the chloride quantified for sodium.

The total fat content was quantified following the Soxhlet (behr ED) method with ether petroleum solvent. The fatty acid profile was determined according to the procedure described by Cruz et al. [20], after the extraction of the fat with nonhalogenated organic solvents, followed by methylation with 0.5 M potassium hydroxide and boron trifluoride in methanol, and separation in a CP-Select FAME chromatographic column (100 m, Agilent, Santa Clara, CA, USA), with adequate calibration standards.

### 2.3. Statistical Analysis

An analysis of variance was performed on meat quality data using the IBM SPSS Statistics Base 22.0 for Windows. Descriptive statistics were generated for all the variables in the dataset. The analysis was carried out using a t-test of independent samples with variable grouping of breast and drumstick portions.

## 3. Results

### 3.1. Carcass Traits Evaluation

The carcass characteristics of the “Barcelos” and “Amarela” roosters are shown in Table 1. The BW and CW showed higher values (*p* ≤ 0.001) in the roosters of the Sasso line than those of the “Amarela” breed, at the same age.

### 3.2. Chemical Composition of the Roosters Raw Meat

The chemical composition of the “Barcelos” and “Amarela” roosters, expressed in fresh meat weight percentage, is shown in Table 2.

The average values were observed between “Barcelos” and “Amarela” roosters with pH (5.80 vs. 5.90), moisture (72.54 vs. 74.60%), ash (1.13 vs. 1.12%), total fat content (4.31 vs. 3.72%), and protein (22.29 vs. 21.62%) values, as a result of the superior carcass weight of the “Barcelos” rooster.

### 3.3. Drumstick and Breast Chemical Composition

Results regarding the proximate composition analysis showed that, in both groups of rooster origin, drumstick meat had higher values for total fat (*p* ≤ 0.001), as well as pH (*p* ≤ 0.001) and protein (*p* ≤ 0.001) contents, when compared to the breast meat (Table 2). “Amarela” roosters presented lower breast moisture (*p* ≤ 0.01) content in comparison with drumstick meat.

There was also higher variability in the protein (breast) and fat (drumstick) contents, which can be attributed to the interaction between genotype and dietary treatment. When compared by genotype, the breast features significant differences in protein (25.15% “Barcelos” rooster vs. 24.55% “Amarela” breed) (*p* ≤ 0.001) and total fat (1.89 vs. 1.90%) (*p* ≤ 0.001). Related to the drumstick, Barcelos” and “Amarela” roosters differed significantly in terms of moisture (71.81 vs. 75.01%) (*p* ≤ 0.05), fat (6.72 vs. 5.42%) (*p* ≤ 0.001), and protein (19.44 vs. 19.70%,respectively) (*p* ≤ 0.001).

### 3.4. Fatty Acid Profile

The composition of the fatty acid rooster profile (Table 3) was expressed in the percentage of total fatty acids. The saturated fatty acids (SFA), monounsaturated fatty acids (MUFA), and polyunsaturated fatty acids (PUFA) content ranged, in the “Barcelos” rooster, from 26.40 to 33.40%, from 29.30 to 44.80%, and from 19.4 to 29.7%, respectively, and, from “Amarela” roosters, within 32.60 and 34.10%, from 39.98 to 45.40% and from 16.01 to 22.42%, respectively.

Concerning SFA, palmitic acid (C16:0) and stearic acid (C18:0) were determined. Regarding MUFA, oleic acid (C18:1) was the most abundant, followed by palmitoleic acid (C16:1), and the least abundant acid was eicosenoic acid (C20:1). Significant differences between genotypes in C16:0 (*p* ≤ 0.001), C18:0 (*p* ≤ 0.001), ∑SFA (*p* ≤ 0.001), C16:1 (*p* ≤ 0.01), C18:1 (*p* ≤ 0.01), ∑MUFA (*p* ≤ 0.001), C18:2n6 (*p* ≤ 0.001), C20:4n6 (*p* ≤ 0.001), ∑n-6-PUFA (*p* ≤ 0.001), C22:5n3 (*p* ≤ 0.01), C22:6n3 (*p* ≤ 0.01), ∑n-3-PUFA (*p* ≤ 0.01), ∑LC-PUFAS (*p* ≤ 0.001), and ∑PUFA (*p* ≤ 0.001) fatty acids, were verified.

C16:0 was the most abundant saturated fatty acid (Table 3), with similar percentages in breast and drumstick portions. Significant differences (*p* ≤ 0.001) for C16:1 and C18:1 were verified, with the drumstick portion having the highest values and for eicosenoic acid (C20:1) (*p* ≤ 0.05), there were higher proportions in the breast portion. For the n-6-PUFA, significant differences (*p* ≤ 0.001) were found in linoleic (C18:2n6), arachidonic (C20:4n6), and adrenic (C22:4n6) acids. However, the first two had a higher proportion in the drumstick, while adrenic acid was more represented in the breast. For n-3-PUFA, the long-chain docosapentaenoic acid (C22:5n3) and docosahexaenoic acid (C22:6n3) were significantly different and were dominant in the breast portions, while α-linolenic (C18:3n3) was superior in the drumstick sample (*p* ≤ 0.01). In general, breast was the meat portion that presented higher values in polyunsaturated fatty acids (∑PUFA, ∑LC-PUFAs, ∑n-3 PUFA, and ∑n-6 PUFA) and saturated (∑SFA) and trans fats, while the drumstick was richer in monounsaturated fatty acids (∑MUFA).

Differences between genotypes, in breast, were observed in C18:0 (*p* ≤ 0.01), ∑SFA (*p* ≤ 0.001), C18:1 (*p* ≤ 0.01), ∑MUFA (*p* ≤ 0.001), C18:2n6 (*p* ≤ 0.001), ∑n-6 PUFA (*p* ≤ 0.001), C22:5n3 (*p* ≤ 0.01), C22:6n3 (*p* ≤ 0.01), ∑n-3-PUFA (*p* ≤ 0.001), ∑LC-PUFAS (*p* ≤ 0.001), and ∑PUFA (*p* ≤ 0.001). Concerning drumstick, they were observed in C16:0 (*p* ≤ 0.01), C18:0 (*p* ≤ 0.01), ∑SFA (*p* ≤ 0.001), C18:2n6 (*p* ≤ 0.001), ∑n-6-PUFA (*p* ≤ 0.001), ∑LC-PUFAS (*p* ≤ 0.05), and ∑PUFA (*p* ≤ 0.001).

## 4. Discussion

The genotype and production system, particularly outdoor production, affect many aspects of the overall quality of poultry products, including nutrient content and functional properties [21,22]. Our results were in accordance with different studies presenting similar genotypes and production systems, such as Sasso line roosters and autochthonous breeds capons and roosters, produced in a traditional system [8,21,22,23,24].

The pH values were similar to those observed in roosters, capons, or other native breeds of chickens, by different authors [25,26]. Values may be affected by different intrinsic factors, such as age (at slaughter), genotype (breed), and sex, with particular influence in the sensorial and technological characteristics of meat [25,26,27].

Lower pH values are related to the production system, extensive or organic, due to better welfare conditions that reduced pre-slaughter stress and, therefore, glycogen consumption. Stress plays a key role in pH rate decline: short-term stress before slaughter increases the in vivo metabolic rate and influences the perimortem metabolism of the animal [21,28].

Differences (*p* ≤ 0.001) in the pH values found in the drumstick and breast could be explained by the unequal activity of each muscle, with a higher concentration of glycogen and less activity in the muscle with lower pH values [29,30]. Animal physical activity can change the metabolic characteristics of the muscles, increasing the muscle oxidative capacity, with the pyruvate aerobic enhancement causing a sparing of glycogen. The hypothetically greater availability of glycogen, and the predominance of oxidative muscles in the drumstick and glycolytic muscles in the breast, could explain the pH values observed [23,29,31].

Moisture is also affected by genotype and slaughtering age [25,32,33]. More moisture could indicate a less physiologically mature state, and concerning the age effect, in general, with increasing age, the level of moisture decreased in meat. A lower moisture percentage observed in our study, compared to broilers or indigenous breeds, may be due to rooster slaughter age (more than 120 days old) and genotype [24,29,34].

Statistically significant differences (*p* ≤ 0.05) in moisture content between drumstick and breast were verified, inside the range of values reported by other authors [24,34,35]. Similar mean values of moisture content in pectoralis major muscle (74%) were obtained, within the range of values described by [29,31,32,33], in autochthonous breeds and improved hybrids commercial breeds for meat production.

The ash content was similar to the ash contents observed in previous studies for Portuguese autochthonous breeds or other indigenous breeds. Other authors present slightly higher ash contents, including in native breeds, and the differences could be due to the lack of standardization in the productive performances and meat quality traits of the animals [23,25,30,33].

Concerning protein, our results were very similar to those described in the literature for autochthonous breeds and broilers [30,33,35,36]. The effect of the genotype was observed, in accordance with different studies with indigenous breeds, not submitted to genetic selection for productive performance and meat quality characteristics, presenting greater variability when compared to commercially used hybrids [33,35,36,37].

Age (tissue protein deposition persisting after 150 days old [31]), genotype, rearing system, and feeding system had a significant effect on muscle development (protein content of drumstick and breast) [25,29,33,38]. Protein content in autochthonous breeds [24,29,32,37,39] or in commercial settings [38,40] reaches, within the range obtained by both cuts, the highest values in the breast, in accordance with our results.

Poultry meat is known for its low fat because, unlike other meat animals, fat is mainly deposited subcutaneously or in the abdomen, rather than in the meat. In the present study, fat content differences were observed between genotypes, with similar values to those in autochthonous breeds [24,33,37,40] or a free-range system [28,41].

According to different authors, lipid content in the muscle is influenced by age and genotype, diet composition, and environment [42,43]. Drumstick meat had significantly more fat than breast meat, which is leaner (typically < 2 g fat/100 g) than other meats and supplies high-quality protein. Breast meat has low-fat content due to the reduced need to store energy [39,43], as observed in our study.

In the second week of life, approximately 36 and 35% of lean meat is located in the breast and legs, respectively, and as chickens grow, the percentage of lean tissue increases to 44% in breast and reduces to 32% in drumsticks, relative to total carcass lean tissue [43,44]. A greater motion reduced the abdominal fat yield of chickens, in the free-range system, and favored muscle mass development [32,45].

Lipids are important constituents of the diet because, in addition to their high energy values, they provide fat-soluble vitamins and essential fatty acids [46]. In an intensive system of mass production, stress increase, and deterioration of broiler carcass quality and meat characteristics. Under a high rearing density, high levels of heat and ammonia are produced and are associated with excessive production of reactive oxygen species, which reduce the immune function and antioxidant activities [47]. Poultry meat has been considered one of the main sources of PUFA, particularly n-3 PUFA, for human diets [47,48].

According to different authors [24,41,49], high levels of polyunsaturated, n-3 PUFA, and SFA total contents are obtained in the breast meat of organically produced animals, slightly higher than those obtained in the present study.

Similar results were observed by [50], concerning saturated, monounsaturated, and PUFA contents of chicken breast meat from conventional and organic farms. Breast meat from birds with free access to pasture presents lower levels of the n-6 and n-3 fatty acid precursors linoleic acid (18:2n-6) and alpha-linolenic acid (18:3n-3), respectively. Fatty acid profile is related to rearing methods [50,51], diet composition, and dietary changes, so farm fodder in organic systems can be used to modify the proportion of PUFA.

“Barcelos” and “Amarela” roosters were characterized by high proportions of individual and total n-3 fatty acids and a favorable n-6/n-3 fatty acid ratio. In autochthonous populations, breed and feeding significantly affect fatty acid content (lower SFA, n-6/n-3 ratio, and higher PUFA and P/S ratio), presenting a favorable fatty acid profile [25,29], as observed in our study. In native breeds, the mechanism implicated in the incorporation of long PUFA in the muscles is more efficient, and the PUFA content depends on its process of elongation/desaturation [24].

The meat of Blackboned chickens had relatively low contents of saturated fatty acids and, in breast muscle, high contents of polyunsaturated fatty acids [40]. Other authors reported SFA [23] as the predominant fatty acid; it exhibits a breed effect on fatty acid composition [52]. The most abundant monounsaturated fatty acid is oleic acid (C18:1), followed by palmitoleic (C16:1) and linoleic (C18:2), and the main polyunsaturated fatty acids are linolenic (C18:3) and araquidonic (C20:4) [53].

## 5. Conclusions

Poultry meat contributes to human nutrition by providing high-quality protein and low levels of fat, with a desirable fatty acid profile, and consumers are increasingly demanding poultry meat products.

To guarantee authenticity, and to protect, qualify, and promote the traditional “Barcelos rooster” dish, chemical and sensorial characterization is mandatory. The influence of the genotype must be considered as a relevant factor in the “Barcelos Rooster” qualification, in particular due to the significantly higher total fat and protein contents, and the favorable n-6/n-3 fatty acid ratio of Sasso line, in comparison with “Amarela” authoctonous rooster bred in some traditional production systems.

After characterizing the production system and obtaining a product similar to that of native animal genotypes, further studies are needed in the sensorial and in the the confection process characterization, with a view to their qualification.

## Figures and Tables

**Table 1:** The carcass characteristics of the “Barcelos” and “Amarela” roosters (average ± SD, Min and Max).

	“Barcelos” Rooster (N = 10)			“Amarela” Rooster (N = 10)			Sig.
Traits	Mean ± SD	Min	Max	Mean ± SD	Min	Max	B*A
**LW(g)**	4211.40 ± 105.22	4035.00	4355.00	3520.20 ± 224.80	3291.00	3835.00	***
**CW1(g)**	3860.40 ± 148.73	3700.00	4005.00	3238.00 ± 259.39	2945.00	3575.00	***
**CW2(g)**	3404.20 ± 149.99	3237.00	3590.00	2941.40 ± 270.97	2606.00	3264.00	***
**CW3(g)**	3072.00 ± 101.89	2915.00	3190.00	2630.00 ± 229.37	2350.00	2900.00	***
**EW(g)**	372.00 ± 14.58	356.00	387.00	311.40 ± 42.08	256.00	364.00	***

SD—standard deviation; Min—minimum; and Max—maximum. LW, live weight at slaughter; CW1, bled and plucked carcass weight; CW2, eviscerated carcass, with head, feet, and edible viscera weight; CW3, eviscerated carcass, without head, feet, and edible viscera weight; and EW, edible viscera weight (head, feet, gizzard, heart, liver, and kidneys). B*A column indicates significant differences for the same parameters between the “Barcelos” and “Amarela” genotypes. *** (*p* ≤ 0.001).

**Table 2:** Effect of breed and rooster pieces, breast, and drumstick in the chemical composition of “Barcelos” and “Amarela” roosters (expressed in % weight of fresh meat) (mean ± SD, Min and Max).

		“Barcelos” Rooster			“Amarela” Rooster			Sig.
		Mean ± SD	Min	Max	Mean ± SD	Min	Max	B*A
**Breast** **(N = 10)**	**pH**	5.68 *** ± 0.16	5.41	5.98	5.78 *** ± 0.13	5.65	5.91	NS
	**Moisture (%)**	73.28 ** ± 1.17	71.41	76.50	73.05 *** ± 1.45	71.60	74.50	NS
	**Ash (%)**	1.15 * ± 0.06	1.03	1.28	1.13 * ± 0.04	1.09	1.17	NS
	**Fat (%)**	1.89 *** ± 1.20	0.16	4.14	1.90 *** ± 1.01	0.89	2.91	***
	**Protein (%)**	25.15 *** ± 1.78	21.96	28.4	24.55 *** ± 0.52	24.03	25.07	***
**Drumstick** **(N = 10)**	**pH**	5.91 ± 0.18	5.70	6.69	5.94 ± 0.05	5.89	6.00	NS
	**Moisture (%)**	71.81 ± 1.86	68.29	74.86	75.0 ± 0.83	74.52	76.0	*
	**Ash (%)**	1.10 ± 0.07	0.99	1.27	1.10 ± 0.06	1.01	1.22	NS
	**Fat (%)**	6.72 ± 1.66	4.35	9.38	5.4 ± 1.31	4.13	6.73	***
	**Protein (%)**	19.44 ± 1.24	16.56	21.70	19.7 ± 0.52	19.13	20.70	***

SD—standard deviation; Min—minimum; Max—maximum. Averages with different symbols in the same column indicate significant differences for the same parameters between the breast and the drumstick of the “Barcelos” and “Amarela” Roosters. B*A column indicates significant differences for the same parameters between the “Barcelos” and “Amarela” genotypes. NS = not significant; *** (*p* ≤ 0.001); ** (*p* ≤ 0.01); and * (*p* ≤ 0.05).

**Table 3:** Fatty acid profile of “Barcelos” and “Amarela” roosters, breast and drumstick portion (results expressed in % of total fatty acids (mean ± SD, Min. and Max).

		“Barcelos” Rooster (N = 10)			“Amarela” Rooster (N = 10)			Sig
**Breast** **(N = 10)**	**Fatty Acid (%)**	**Mean** **± SD**	**Min**	**Max**	**Mean** **± SD**	**Min**	**Max**	B*A
	C16:0	21.34 ± 1.76	17.80	24.10	23.02 ± 0.59	22.43	23.61	NS
	C18:0	7.63 ± 0.68	6.30	8.70	6.45 ± 0.53	5.92	6.98	**
	**⅀SFA**	**30.30 ± 1.61**	**27.60**	**33.40**	**33.2 ± 0.60**	**32.60**	**33.80**	***
	C16:1	3.85 *** ± 0.96	2.20	5.30	3.55 *** ± 0.31	3.24	3.86	NS
	C18:1	31.91 *** ± 2.72	26.00	36.10	33.10 *** ± 1.12	31.98	34.22	**
	C20:1	0.47 * ± 0.08	0.30	0.60	0.42 ± 0.08	0.34	0.50	NS
	**⅀MUFA**	36.74 *** ± 3.57	**29.30**	**41.80**	41.08 *** ± 1.10	**39.98**	**42.18**	***
	C18:2n6	17.02 ** ± 2.05	14.10	21.60	14.15 *** ± 0.55	13.60	14.70	***
	C20:4n6	4.88 *** ± 2.48	2.00	10.40	4.95 *** ± 0.81	4.14	5.76	NS
	C22:4n6	0.52 *** ± 0.2	0.30	0.90	0.63 *** ± 0.13	0.50	0.76	NS
	⅀n-6-PUFA	22.75 ± 2.43	18.60	25.90	20.1 ± 2.23	17.87	22.33	**
	C18:3n3	0.96 ** ± 0.22	0.70	1.50	1.06 *** ± 0.05	0.41	0.51	NS
	C22:5n3	0.66 *** ± 0.39	0.30	1.70	0.48 ** ± 0.08	0.40	0.56	**
	C22:6n3	0.74 *** ± 0.33	0.30	1.40	0.42 ** ± 0.06	0.36	0.48	**
	⅀n-3-PUFA	2.47 *** ± 0.55	1.80	3.90	1.96 ± 0.68	0.80	2.16	***
	⅀LC-PUFAS	1.99 *** ± 0.88	1.00	4.10	1.56 ** ± 0.81	0.75	2.37	***
	**⅀PUFA**	**25.21 ± 2.81**	**20.60**	**29.70**	21.10 *** ± 1.25	**19.90**	**22.42**	***
	TRANS	2.57 ** ± 0.30	2.10	3.20	2.51 ± 0.26	2.25	2.77	NS
**Drumstick** **(N = 10)**	C16:0	21.33 ± 1.60	19.10	24.10	22.87 ± 0.42	22.45	23.29	**
	C18:0	7.24 ± 0.77	6.00	8.60	6.15 ± 0.65	5.50	6.80	**
	**⅀SFA**	**29.68 ± 1.96**	**26.40**	**33.30**	**33.50 ± 0.55**	33.00	34.10	***
	C16:1	5.19 ± 0.98	6.60	5.19	6.89 ± 0.25	6.64	7.14	*
	C18:1	35.19 ± 1.63	32.30	37.30	35.78 ± 0.42	35.36	36.20	NS
	C20:1	0.41 ± 0.06	0.30	0.50	0.37 ± 0.05	0.32	0.42	NS
	**⅀MUFA**	**41.45 ± 2.25**	**38.20**	**44.80**	**44.30 ± 1.12**	**43.20**	**45.40**	***
	C18:2n6	19.41 ± 2.14	15.10	22.50	17.19 ± 0.36	16.83	17.55	***
	C20:4n6	2.13 ± 0.65	1.40	3.40	2.12 ± 0.19	1.93	2.31	NS
	C22:4n6	0.30 ± 0.08	0.20	0.40	0.42 ± 0.14	0.28	0.56	NS
	⅀n-6-PUFA	22.18 ± 2.36	17.90	25.10	20.1 ± 2.12	17.98	22.22	***
	C18:3n3	1.15 ± 0.19	0.90	1.50	1.20 ± 0.21	0.99	1.41	NS
	C22:5n3	0.25 ± 0.10	0.10	0.50	0.32 ± 0.11	0.21	0.43	NS
	C22:6n3	0.24 ± 0.09	0.10	0.40	0.28 ± 0.10	0.18	0.38	NS
	⅀n-3-PUFA	1.75 ± 0.26	1.20	2.10	1.91 ± 0.08	1.83	1.99	NS
	⅀LC-PUFAS	0.87 ± 0.22	0.60	1.30	1.15 ± 0.15	1.00	1.30	*
	**⅀PUFA**	**23.94 ± 2.49**	**19.40**	**27.30**	**17.3 ± 1.32**	**16.01**	**18.62**	***
	TRANS	2.32 ± 0.23	2.00	2.80	2.28 ± 0.26	2.02	2.54	NS

SD—standard deviation; Min—minimum; Max—maximum. SFA—saturated fatty acids; MUFA—monounsaturated fatty acids; and PUFA—polyunsaturated fatty acids. Averages with different symbols in the same column indicate significant differences for the same parameters between the breast and the drumstick of the “Barcelos” and “Amarela” Roosters. B*A column indicates significant differences for the same parameters between the “Barcelos” and “Amarela” genotypes. NS = not significant; *** (*p* ≤ 0.001); ** (*p* ≤ 0.01); and * (*p* ≤ 0.05).

## Data Availability

The raw data have been submitted to CISAS-IPVC (Center for Research and Development in Agrifood Systems and Sustainability—Polytechnic Institute of Viana do Castelo) and are available on request.
